# Hypertriglyceridemic Waist Might Be an Alternative to Metabolic Syndrome for Predicting Future Diabetes Mellitus

**DOI:** 10.1371/journal.pone.0073292

**Published:** 2013-09-05

**Authors:** Sen He, Yi Zheng, Yan Shu, Jiyun He, Yong Wang, Xiaoping Chen

**Affiliations:** 1 Department of Cardiovascular Medicine, West China Hospital, Sichuan University, Chengdu, China; 2 Department of Cardiovascular Medicine, Sichuan Provincial People’s Hospital, Chengdu, China; Fundación para la Prevención y el Control de las Enfermedades Crónicas No Transmisibles en América Latina (FunPRECAL), Argentina

## Abstract

**Background:**

In some cross-sectional studies, hypertriglyceridemic waist (HTGW) has been recommended as an alternative to metabolic syndrome (MetS) for screening individuals at high risk for diabetes mellitus (DM). However, little information is about the predictive power of HTGW for future DM. The aims of the study were to assess the DM predictive power of HTGW compared with MetS based on the follow-up data over 15 years collected from a general Chinese population.

**Methods:**

**And Findings**: The data were collected in 1992 and then again in 2007 from the same group of 687 individuals without DM in 1992. For the whole population (n =687), multivariate analysis showed presence of HTGW was associated with a 4.1-fold (95%CI: 2.4-7.0, *p* < 0.001) increased risk and presence of MetS was associated with a 3.7-fold (95%CI: 2.2-6.2, *p* < 0.001) increased risk for future DM. For the population without elevated fasting plasma glucose (n = 650), multivariate analysis showed presence of HTGW was associated with a 3.9-fold (95%CI: 2.2-7.0, *p* < 0.001) increased risk and presence of MetS was associated with a 3.7-fold (95%CI: 2.1-6.6, *p* < 0.001) increased risk for future DM.

**Conclusions:**

HTGW could predict future DM independently, and the predictive power was similar to MetS. HTGW might be an alternative to MetS for predicting future DM. For simpler and fewer components, HTGW might be more practical than MetS, and it might be recommended in most clinical practices. This finding might be more useful for the individuals who only have elevated WC and TG. Although these individuals are without MetS, they are still at high risk for future DM, similarly to the individuals with MetS.

## Introduction

The number of individuals with diabetes mellitus (DM) worldwide has more than doubled over the past three decades, and it has been predicted that the number of diabetic patients would increase to 439 million by 2030 [1]. DM and related-complications have been recognized as a major global public health problem [1]. Fighting with DM, prevention is the best intervention. Identifying individuals who have a high risk for DM is potentially of significant benefit if preventive measures are used. It is well known that metabolic syndrome (MetS) is a useful screening tool to identify individuals at high risk for DM in clinical practices [2], which includes five components, such as dysglycemia, raised blood pressure, elevated triglyceride levels, low high-density lipoprotein cholesterol levels and obesity (particularly central adiposity) [2].

Recently, hypertriglyceridemic waist (HTGW), which means elevated triglyceride levels and enlarged waist circumstance, has also been recommended as a useful tool for screening individuals at high risk for DM in some cross-sectional studies [3–10], even as an alternative to MetS [3,11–13]. However, little information is about the predictive power of HTGW for future DM, as compared with MetS. Therefore, the main aims of our study were to assess the DM predictive power of HTGW compared with MetS based on the follow-up data over 15 years collected from a general Chinese population.

## Methods

### Study population

The data were collected in 1992 and then again in 2007 from the same group of 711 participants in an urban community located in Chengdu, Sichuan province, China. Detailed information of these participants has been reported elsewhere [14–16]. Since 24 participants were diagnosed with DM in 1992, they were excluded from the analysis. Therefore, only 687 participants with complete data were available and analysed. This study was approved by Ministry of Health of China, as well as by the Ethics Committee of West China Hospital of Sichuan University. All participants provided written informed consent.

### Related definitions

MetS was defined as the new joint interim statement [2], and the presence of any 3 of 5 aftermentioned risk factors constituted a diagnosis of MetS: (1) elevated TG was defined as 1.7 mmol/L or greater; (2) elevated blood pressure (BP) was defined as systolic BP (SBP) ≥ 130 and/or diastolic BP (DBP) ≥ 85 mmHg and/or those receiving antihypertensive medications; (3) reduced high-density lipoprotein cholesterol (HDL-C) was defined as a level less than 1.0 mmol/L for men and a level less than 1.3 mmol/L for women; (4) elevated fasting plasma glucose (FPG) was defined as 5.6 mmol/L or greater; (5) for asians, elevatedWC was defined as 80 cm or greater for women and 90 cm or greater for men [2,17]. HTGW was defined as WC of at least 90 cm for men or at least 80 cm for women together with a TG level of at least 1.7 mmol/L. DM was defined by self-reported history or a FPG ≥ 7.0 mmol/L. Smoking: average cigarette consumption ≥ one/day. Alcohol intake: average intake of alcohol ≥ 50 g/day. Physical activity: exercise one or more times per week, at least 20 minutes for each time.

### Statistical analysis

Data are presented as means ± standard deviation (SD) for normally continuous variables, or median + inter-quartile range for skewedly continuous variables. Categorical variables were used as dummy variables (presence = 1, absence = 0). Independent t test and non-parametric test (Mann-Whitney test) were used where appropriate to compare continuous variables. Interactions between categorical variables were evaluated with the Pearson χ^2^ test. COX proportional hazards models were used to estimate hazard ratios (HRs) of incident DM associated with HTGW or MetS. For statistical analysis, the SPSS software package (version 17.0; SPSS, Chicago, IL) was used. Statistical significance was defined as *p* < 0.05.

## Results

### Analyses of the whole population (n = 687)

In general, individuals with either HTGW or MetS had higher levels of CVD risk factors compared with their respective control group, such as age, SBP, DBP, total cholesterol (TC), TG, body mass index (BMI) and WC (Table 1). No statistically significant differences were also found in some other CVD risk factors such as low-density lipoprotein cholesterol (LDL-C), alcohol intake, physical activity and family history of DM. Like the data at baseline, the data in 2007 also showed that individuals with either HTGW or MetS had higher levels of CVD risk factors compared with their respective control group (data not shown).

**Table 1:** Efficacy of hypertriglyceridemic waist and metabolic syndrome in identifying individuals with metabolic abnormalities among the whole population at baseline.

Variables	Non-HTGW (n = 606)	HTGW (n = 81)	*p* value*	Non-MetS (n = 590)	MetS (n = 97)	*p* value†
Age (years)	47.8 ± 6.2	50.3 ± 6.2	0.001	47.7 ± 6.1	50.7 ± 6.6	< 0.001
Male sex	373 (61.6)	26 (32.1)	< 0.001	365 (61.9)	34 (35.1)	< 0.001
SBP (mmHg)	110.0 (104.0, 120.0)	122.2 ± 18.2	< 0.001	110.0 (104.0, 120.0)	129.2 ± 19.9	< 0.001
DBP (mmHg)	71.0 (70.0, 80.0)	79.0 (70.0, 82.0)	< 0.001	70.0 (70.0, 78.0)	80.0 (73.5, 89.0)	< 0.001
FPG (mmol/L)	4.2 (3.8, 4.7)	4.2 (3.8, 4.9)	0.505	4.0 (3.8, 4.7)	4.5 (4.0, 5.2)	0.001
TC (mmol/L)	4.4 (3.9, 5.0)	4.7 ± 0.9	0.018	4.3 (3.9, 4.9)	4.7 ± 0.9	0.002
TG (mmol/L)	1.8 (1.4, 2.3)	2.4 (2.1, 3.3)	< 0.001	1.8 (1.4, 2.2)	2.3 (2.1, 3.3)	< 0.001
LDL-C (mmol/L)	2.3 ± 0.8	2.2 ± 1.1	0.433	2.3 ± 0.8	2.3 ± 1.0	0.869
HDL-C (mmol/L)	1.2 (1.1, 1.4)	1.2 ± 0.2	0.154	1.3 (1.1, 1.4)	1.1 (1.0, 1.3)	< 0.001
BMI (kg/m^2^)	22.9 ± 2.6	26.8 ± 2.2	< 0.001	23.0 ± 2.6	25.8 ± 2.9	< 0.001
WC (cm)	75.0 (70.0, 80.0)	86.7 ± 5.7	< 0.001	75.0 (70.0, 80.0)	82.6 ± 7.9	< 0.001
Elevated WC	22 (3.6)	81 (100.0)	< 0.001	43 (7.3)	60 (61.9)	< 0.001
Elevated TG	319 (52.6)	81 (100.0)	< 0.001	304 (51.5)	96 (99.0)	< 0.001
Elevated BP	119 (19.6)	29 (35.8)	0.001	86 (14.6)	62 (63.9)	< 0.001
Reduced HDL-C	204 (33.7)	42 (51.9)	0.001	169 (28.6)	77 (79.4)	< 0.001
Elevated FPG	28 (4.6)	9 (11.1)	0.015	17 (2.9)	20 (20.6)	< 0.001
HTGW	N/A	N/A	N/A	22 (3.7)	59 (60.8)	< 0.001
Smoking	231 (38.1)	17 (21.0)	0.003	231 (39.2)	17 (17.5)	< 0.001
Alcohol intake	81 (13.4)	6 (7.4)	0.130	78 (13.2)	9 (9.3)	0.279
Physical activity	132 (21.8)	14 (17.3)	0.353	127 (21.5)	19 (19.6)	0.665
Family history of DM	22 (3.6)	4 (4.9)	0.562	22 (3.7)	4 (4.1)	0.850
MetS	38 (6.3)	59 (72.8)	< 0.001	N/A	N/A	N/A

^*^Between Non-HTGW and HTGW; † between Non-MetS and MetS.Data are presented as means ± SD, or median (inter-quartile range), or number (percentage). SBP = systolic blood pressure; DBP = diastolic blood pressure; FPG = fasting plasma glucose; TC = serum total cholesterol; TG = triglyceride; LDL-C = low-density lipoprotein cholesterol; HDL-C = high-density lipoprotein cholesterol; BMI = body mass index; WC = waist circumference; BP = blood pressure; HTGW = hypertriglyceridemic waist; MetS = metabolic syndrome; DM = diabetes mellitus.

**Figure 1:**
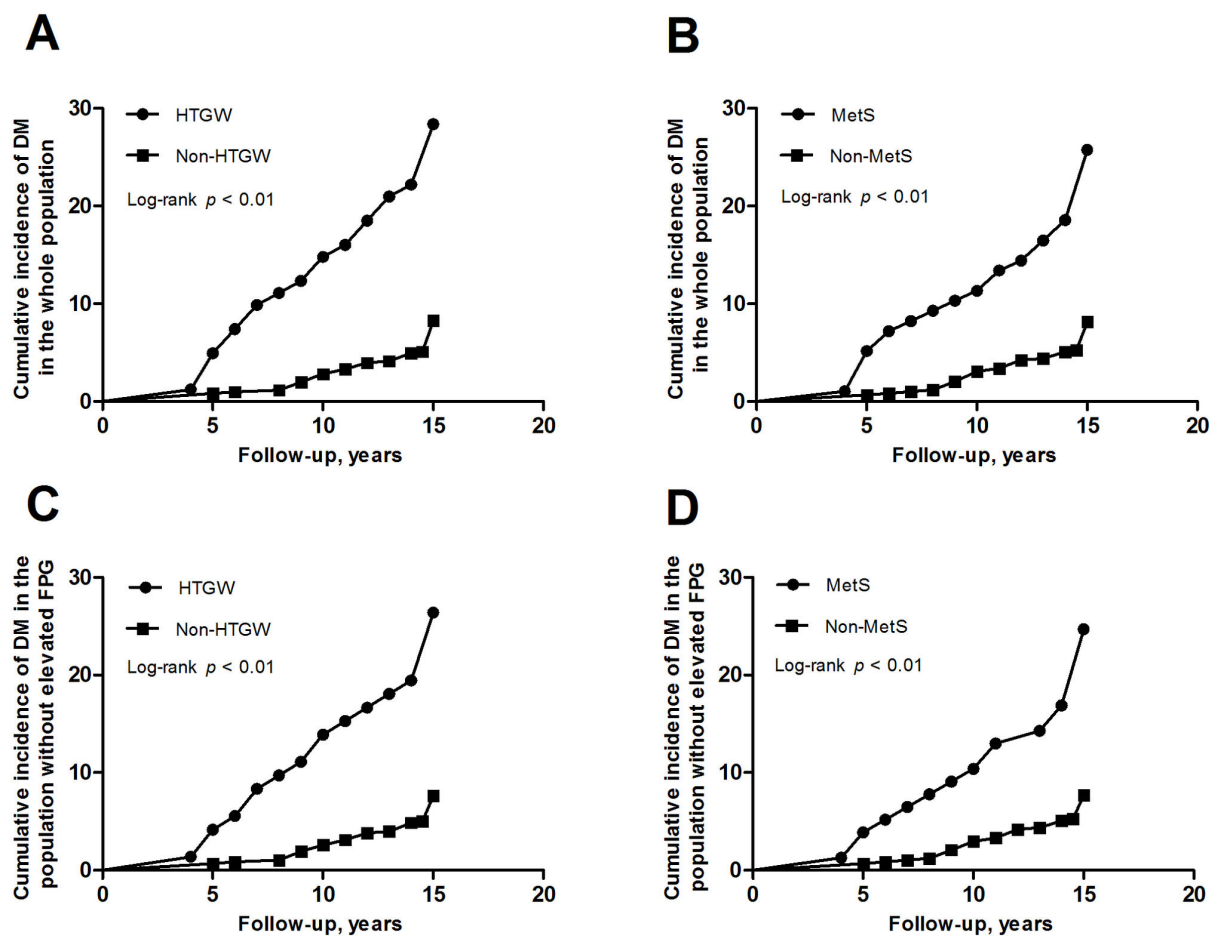
Cumulative incidence of diabetes mellitus by presence or absence of metabolic abnormalities at baseline in different population. (A): presence or absence of HTGW in the whole population; (B): presence or absence of MetS in the whole population; (C): presence or absence of HTGW in the population without elevated FPG; (D): presence or absence of MetS in the population without elevated FPG. DM = diabetes mellitus; HTGW = hypertriglyceridemic waist; MetS = metabolic syndrome; FPG = fasting plasma glucose.

Unadjusted cumulative incidence of DM from 1992 and 2007 according to baseline HTGW or MetS is shown in Figure 1. The cumulative incidence was higher in the group which had HTGW than the group without HTGW (28.4% vs. 8.4%, log-rank *p* < 0.001) (Figure 1A). The cumulative incidence was also higher in the group which had MetS than the group without MetS (25.8% vs. 8.3%, log-rank *p* < 0.001) (Figure 1B). The cumulative incidence of DM was not different between the group of HTGW and the group of MetS (28.4% vs. 25.8%, *p* > 0.05), and similar result was also found between the other two groups (8.4% vs. 8.3%, *p* > 0.05).

The univariate COX regression analysis presented that HTGW could predict future DM independently (HR: 3.9, 95%CI: 2.4-6.3, *p* < 0.001), as well as MetS (HR: 3.4, 95%CI: 2.1-5.6, *p* < 0.001) (Table 2). After adjustment for confounders, HTGW could still predict future DM (HR: 4.1, 95%CI: 2.4-7.0, *p* < 0.001) (Table 2). Because elevated BP, reduced HDL-C and elevated FPG are components of MetS, we excluded them from HR estimation of incident DM associated with MetS for avoiding excessive adjustments. For MetS, the HR was 3.7 (95%CI: 2.2-6.2, *p* < 0.001), which was similar to HTGW (Table 2).

**Table 2:** Univariate and multivariate COX regression models for predicting future diabetes mellitus in the whole population (n = 687).

Variables	Univariate analysis			Model 1			Model 2	
	HR (95%CI)	*p* value		HR (95%CI)	*p* value		HR (95%CI)	*p* value
Age (years)	1.0 (1.0-1.1)	0.014		1.0 (1.0-1.1)	0.378		1.0 (1.0-1.1)	0.300
Sex (1 = male, 2 = female)	0.7 (0.5-1.2)	0.224		0.5 (0.3-1.1)	0.070		0.7 (0.4-1.4)	0.347
TC (mmol/L)	1.3 (1.0-1.7)	0.041		1.2 (0.9-1.6)	0.138		1.2 (0.9-1.6)	0.172
Elevated WC	3.2 (2.0-5.2)	< 0.001						
Elevated TG	2.2 (1.3-3.6)	0.004						
Elevated BP	1.7 (1.0-2.8)	0.036		1.3 (0.8-2.1)	0.377			
Reduced HDL-C	1.5 (0.9-2.3)	0.099		1.8 (1.1-2.9)	0.026			
Elevated FPG	3.0 (1.5-5.8)	0.001		2.5 (1.2-4.9)	0.011			
Smoking	1.6 (0.9-2.2)	0.193		1.5 (0.8-2.8)	0.174		1.5 (0.8-2.7)	0.193
HTGW	3.9 (2.4-6.3)	< 0.001		4.1 (2.4-7.0)	< 0.001			
MetS	3.4 (2.1-5.6)	< 0.001					3.7 (2.2-6.2)	< 0.001

TC = serum total cholesterol; WC = waist circumstance; TG = triglyceride; BP = blood pressure; HDL-C = high-density lipoprotein cholesterol; FPG = fasting plasma glucose; DM = diabetes mellitus; HTGW = hypertriglyceridemic waist; MetS = metabolic syndrome; CI = confidence interval; HR = hazard ratio. Model 1 included age, sex, TC, elevated BP, reduced HDL-C, elevated FPG, smoking and HTGW; Model 2 included age, sex, TC, smoking and MetS.

### Analyses of the subgroup without elevated fasting plasma glucose (n = 650)

It is well known that the individuals with elevated FPG are at higt risk for future DM, and we conducted some additional analyses of the population without elevated FPG (n = 650). In this subgroup, both HTGW and MetS could identify the individuals with metabolic abnormalities in 1992 and 2007 (data not shown).

Unadjusted cumulative incidence of DM from 1992 and 2007 according to baseline HTGW or MetS is shown in Figure 1. The cumulative incidence was higher in the group which had HTGW than the group without HTGW (26.4% vs. 7.8%, log-rank *p* < 0.001) (Figure 1C). The cumulative incidence was also higher in the group which had MetS than the group without MetS (24.7% vs. 7.9%, log-rank *p* < 0.001) (Figure 1D). The cumulative incidence of DM was not different between the group of HTGW and the group of MetS (26.4% vs. 24.7%, *p* > 0.05), and similar result was also found between the other two groups (7.8% vs. 7.9%, *p* > 0.05).

The univariate COX regression analysis presented that HTGW could predict future DM independently, as well as MetS (Table 3). After adjustment for confounders, both HTGW and MetS could still predict future DM, and the HRs were 3.9 (95%CI: 2.2-7.0, *p* < 0.001) and 3.7 (95%CI: 2.1-6.6, *p* < 0.001) respectively (Table 3).

**Table 3:** Univariate and multivariate COX regression models for predicting future diabetes mellitus in the population without elevated fasting plasma glucose (n = 650).

Variables	Univariate analysis			Model 1			Model 2	
	HR (95%CI)	*p* value		HR (95%CI)	*p* value		HR (95%CI)	*p* value
Age (years)	1.1 (1.0-1.1)	0.011		1.0 (1.0-1.1)	0.191		1.0 (1.0-1.1)	0.195
Sex (1 = male, 2 = female)	0.7 (0.4-1.1)	0.130		0.5 (0.3-1.1)	0.089		0.7 (0.3-1.3)	0.262
TC (mmol/L)	1.3 (1.0-1.7)	0.087		1.2 (0.9-1.7)	0.161		1.2 (0.9-1.6)	0.213
Elevated WC	3.1 (1.9-5.3)	< 0.001						
Elevated TG	2.3 (1.3-4.0)	0.004						
Elevated BP	1.9 (1.1-3.2)	0.017		1.5 (0.8-2.5)	0.172			
Reduced HDL-C	1.4 (0.9-2.4)	0.142		1.8 (1.1-3.0)	0.032			
Smoking	1.4 (0.9-2.3)	0.169		1.5 (0.8-2.8)	0.226		1.5 (0.8-2.7)	0.251
HTGW	3.8 (2.2-6.5)	< 0.001		3.9 (2.2-7.0)	< 0.001			
MetS	3.4 (2.0-5.9)	< 0.001					3.7 (2.1-6.6)	< 0.001

TC = serum total cholesterol; WC = waist circumstance; TG = triglyceride; BP = blood pressure; HDL-C = high-density lipoprotein cholesterol; DM = diabetes mellitus; HTGW = hypertriglyceridemic waist; MetS = metabolic syndrome; CI = confidence interval; HR = hazard ratio. Model 1 included age, sex, TC, elevated BP, reduced HDL-C, elevated FPG, smoking and HTGW. Model 2 included age, sex, TC, smoking and MetS.

## Discussion

The main aims of our study were to assess the DM predictive power of HTGW compared with MetS based on the follow-up data over 15 years collected from a general Chinese population. The results showed that HTGW could predict future DM independently, similarly to MetS, and HTGW might be an alternative to MetS to detect the individuals at high risk for future DM.

It is well known that MetS is associated with DM [2,18–20]. While, is diagnosing metabolic syndrome a uniquely simple way to predict incident type 2 diabetes mellitus [21]? Recently, for the excellent ability of identifying individuals at hight risk of metabolic abnormalities [3,4], HTGW has also been recommended as a marker for screening individuals at high risk for DM in some cross-sectional studies [3–10], even an alternative to MetS [3,11–13]. For example, in a study by Lemieux et al [6], the results showed that a 12-fold increase in the prevalent odds ratio of having DM (95% CI: 5.1-27.9, *p* < 0.001) was observed in men with HTGW compared with the reference group of men with both low WC and TG. Data from NHANES III [7] suggested that individuals with HTGW had a higher prevalence of DM (25.4% vs. 8.0%, *p* < 0.001; RR: 3.2, 95% CI: 2.4-4.0). A cross-sectional study from Canada [8] showed that HTGW was a strong predictor of DM (OR: 8.6, 95%CI: 2.1-34.6) in Inuit, with adjusting for age, sex, region, family history of DM, education and use of lipid-lowering medications.

Prospective study about the association of HTGW with DM is few. To our knowledge, there is only one prospective study that found measurement of WC in combination with TG could improve early screening for gestational glucose intolerance, and the risk remained significant even after controlling for maternal age, fasting glucose at first trimester and previous history of gestational diabetes (OR: 4.7, *p* = 0.02) [22]. In line with this study, our results showed HTGW could predict future DM in the whole population independently. On the other hand, individuals with elevated FPG have been referred to as having pre-diabetes, indicating the relatively high risk for the future development of DM [23], and we excluded the individuals with elevated FPG from the whole population in the subgroup analyses. The results also showed HTGW could predict future DM in the subgroup independently. HTGW could predict future DM independently, which might be explained HTGW are closely related to visceral adipocytes and metabolic abnormalities [3–10,24]. More importantly, our results showed that HTGW had similar predictive power to MetS for future DM, and HTGW might be an alternative to MetS to detect the individuals at high risk for future DM. This finding might be more useful for the individuals who have elevated WC and TG, not having elevated BP, reduced HDL-C and elevated FPG. Although these individuals are without MetS, they are still at high risk for future DM, similarly to the individuals with MetS.

Some potential limitations of this study should be mentioned. First, the absence of an oral glucose tolerance test means that some individuals would have developed DM that was not detectable by changes in fasting glucose alone or by clinical history. However, oral glucose tolerance tests were not feasible for pragmatic reasons. Second, we lacked the information about the drugs used which might influence the levels of serum lipids and the risk for subsequent DM, and long term usage of these drugs could influence our results. Usually, the individuals take medicine erratically in China, so that mightn’t influence the results in our study.

In summary, the results obtained in this prospective study show that HTGW could predict future DM independently, and the predictive power is similar to MetS. HTGW might be an alternative to MetS to detect the individuals at high risk for future DM. For simpler and fewer components, HTGW might be more practical than MetS, and it might be recommended in most clinical practices. This finding might be more useful for the individuals who only have elevated WC and TG. Although these individuals are without MetS, they are still at high risk for future DM, similarly to the individuals with MetS. Future researches may be warranted to assess the predictive power for future DM in different races and larger cohorts.
